# Efficacy of a dynamic collimator for overranging dose reduction in a second- and third-generation dual source CT scanner

**DOI:** 10.1007/s00330-017-4745-8

**Published:** 2017-01-26

**Authors:** Ronald Booij, Marcel L. Dijkshoorn, Marcel van Straten

**Affiliations:** Department of Radiology and Nuclear Medicine, Erasmus MC, P.O. Box 2240, 3000 CA Rotterdam, The Netherlands

**Keywords:** Tomography, spiral computed, Health physics, Radiation dosage, Diagnostic imaging, Phantoms, imaging

## Abstract

**Objectives:**

The purpose of this study was to assess the efficacy of the renewed dynamic collimator in a third-generation dual source CT (DSCT) scanner and to determine the improvements over the second-generation scanner.

**Methods:**

Collimator efficacy is defined as the percentage overranging dose in terms of dose–length product (DLP) that is blocked by the dynamic collimator relative to the total overranging dose in case of a static collimator. Efficacy was assessed at various pitch values and different scan lengths. The number of additional rotations due to overranging and effective scan length were calculated on the basis of reported scanning parameters. On the basis of these values, the efficacy of the collimator was calculated.

**Results:**

The second-generation scanner showed decreased performance of the dynamic collimator at increasing pitch. Efficacy dropped to 10% at the highest pitch. For the third-generation scanner the efficacy remained above 50% at higher pitch. Noise was for some pitch values slightly higher at the edge of the imaged volume, indicating a reduced scan range to reduce the overranging dose.

**Conclusions:**

The improved dynamic collimator in the third-generation scanner blocks the overranging dose for more than 50% and is more capable of shielding radiation dose, especially in high pitch scan modes.

***Key points*:**

• *Overranging dose is to a large extent blocked by the dynamic collimator*

• *Efficacy is strongly improved within the third*-*generation DSCT scanner*

• *Reducing the number of additional rotations can reduce overranging with increased noise*

## Introduction

Spiral computed tomography (CT) has proven its superiority over sequential CT in routine clinical practice. A downside of spiral CT, particularly at an increased detector width and higher pitch values, is the increase of the overranging effect, resulting in a higher dose to the patient [1–4]. Overranging dose is defined as primary radiation that is given to the patient outside the imaged volume [5–7]. The dose penalty due to overranging relative to the total patient dose increases with shorter scan lengths as in paediatrics, the coronary arteries or head and neck imaging [8–10].

In order to reduce the overranging dose, manufacturers introduced dynamic or adaptive collimators to block the dose which is irrelevant for image reconstruction [11–13]. Dynamic collimators are mechanical blades which move in and out of the radiation area to block the irrelevant radiation. With the introduction of a third-generation DSCT scanner, the speed of the blade movement of the dynamic collimator was improved compared to the second-generation DSCT scanner. To our knowledge, no literature is available on the performance of dynamic collimators in state-of-the-art DSCT scanners. Since DSCT is often used at high scan speeds, it is important to be aware of the impact of overranging dose especially in protocols with short scan ranges in (high) radiation-sensitive organs, where overranging can contribute to a larger dose.

The purpose of this study was to assess the efficacy of the renewed dynamic collimator of the third-generation DSCT scanner and compare it to the second-generation DSCT scanner. This was examined by determining the amount and nature of the overranging dose as a function of pitch and scan length.

## Materials and methods

### Scanners, phantom and scanning protocols

Overranging dose was assessed for a second- and third-generation DSCT scanner. The software version of the second-generation DSCT scanner (SOMATOM Definition Flash; Siemens Healthcare, Forchheim, Germany) was Syngo CT 2012B and that of the third-generation DSCT scanner (SOMATOM Force; Siemens Healthcare, Forchheim, Germany) was Syngo CT VA50A.

A 32-cm-diameter CTDI phantom of 15 cm length was positioned at the isocentre of the scanner. Scans started at the centre of the phantom in order to assess the image quality at the edge of the imaged volume in a homogenous object.

Scans were made at various pitch values with a thorax protocol. The pitch values used in single source mode were 0.35, 0.7 and 1.4. Using the dual source mode, the pitch values were 1.55 and 3.2. In addition to the thorax protocol, scans were made with a dedicated dual source mode cardiac ECG gated protocol at pitch 3.4 and pitch 3.2 on the second- and third-generation scanner, respectively.

The other scanning parameters were 120 kVp tube voltage for both tubes, and a combined fixed tube load for both tubes of 100 effective milliampere second (eff. mAs). For the second-generation DSCT scanner the rotation time was 0.285 s and the beam collimation was 64 × 0.6 mm. For the third-generation DSCT scanner a rotation time of 0.25 s was used and a beam collimation of 96 × 0.6 mm. All scans were made at three distances between the first and last reconstructable slice position: 100, 200 and 300 mm. For each scan the DICOM radiation dose structured report (RDSR) was stored.

### Collimator efficacy

The collimator efficacy is defined as the percentage of overranging dose in terms of the dose–length product (DLP) that is blocked by the dynamic collimator relative to the total overranging dose in case of a static open collimator. The efficacy is derived from information available in the DICOM RDSR.

The overranging scan length *L*_o,scan_ is defined as the length of the actual scan range outside the range of the reconstructable volume [14]:

$$ {L}_{\mathrm{o},\mathrm{scan}}={L}_{\mathrm{scan}}-{L}_{\mathrm{r}} $$

where *L*_scan_ is the reported scan range and *L*_r_ is the length of the reconstructable volume [15], i.e. the distance between the first and last reconstructed slice position plus the nominal value of the largest possible slice thickness. The nominal slice thickness is used instead of a value based on measurements of the slice sensitivity profile (SSP) because unambiguous measurements are complicated in the context of this study. The SSP might depend on the position in the axial plane and on the position of the axial slice in the imaged volume. The latter possible dependency was investigated by noise measurements (see “Image reconstruction and noise measurements”).

The number of additional scan rotations *N*_o,scan_ due to overranging is calculated by

$$ {N}_{\mathrm{o},\mathrm{scan}}=\frac{L_{\mathrm{o},\mathrm{scan}}}{M\cdot S\cdot p} $$

where *M* is the single detector row width, *S* is the number of detector rows and *p* is the pitch.

Thanks to the dynamic collimator, the additional dose due to overranging is smaller than one would expect from the additional scan length. The effective overranging length *L*_o,dose_ associated with the increase of the DLP due to overranging is derived from the reported DLP and CTDI_vol_ values for a given reconstructable volume length *L*_r_:

$$ {L}_{\mathrm{o},\mathrm{dose}}=\frac{\mathrm{DLP}}{{\mathrm{CTDI}}_{\mathrm{vol}}}-{L}_{\mathrm{r}} $$

Finally, the efficacy *E* of the dynamic collimator is calculated as follows:

$$ E=\frac{L_{\mathrm{o},\mathrm{scan}}-{L}_{\mathrm{o},\mathrm{dose}}}{L_{\mathrm{o},\mathrm{scan}}}\cdot 100\%. $$

The uncertainties in the calculated values for *N*_o,scan_, *L*_o,dose_ and *E* depend on both the accuracy and precision of the underlying variables CTDI_vol_, DLP, *L*_scan_ and *L*_r_. Repeated scans proved that these variables are very precisely reported in the DICOM RDSR. The precision is limited only by the number of significant figures of the data representation in the RDSR. The imprecision in *E* is therefore estimated by propagation of the imprecisions in the underlying variables CTDI_vol_, DLP, *L*_scan_ and *L*_r_. The inaccuracy of the underlying variables and its influence on the uncertainties in the calculated values were negligible as determined below.

The length *L*_r_ of the reconstructable volume was assumed to be highly accurate. The accuracy of the reported scan range *L*_scan_ was checked by comparing the corresponding scan time with scan time measurements made with an ionization chamber. Preliminary experiments confirmed that the reported scan times are equal to the total time the x-ray tube is on and thus that the reported scan range is equal to to the actual scan range.

It is known that the reported CTDI_vol_ value might deviate as much as 30% from the true dose value. The scanner software calculates the DLP value via multiplication of the reported CTDI_vol_ value by the effective scan length. It is safe to assume that the error in the CTDI_vol_ value is independent of the error in the effective scan length. Therefore, the errors in the CTDI_vol_ value and DLP value correlate, and the error in the CTDI_vol_ cancels out in the calculation of *L*_o,dose_ because the CTDI_vol_ value and DLP value appear in the denominator and numerator of the same fraction, respectively. Consequently, any deviation of the reported CTDI_vol_ value from the true dose value does not affect the calculation of the efficacy *E*.

An inaccurate estimation of the effective scan length by the scanner software results in an inaccurately reported DLP value. Therefore, the accuracy of this effective scan length was checked by comparison of reported DLP values with measured DLP values for various pitch values and scan lengths. Preliminary experiments showed a coefficient of determination *R* ^2^ of 1.00. Therefore, it was assumed that the reported DLP values accurately reflect any change in effective scan length and no additional DLP measurements were performed.

### Image reconstruction and noise measurements

All axial images were reconstructed with a slice thickness of 0.6 mm, 3.0 mm and 10 mm (minimum, mid and maximum slice width reconstruction) using a standard kernel (second-generation scanner, B30f; third-generation scanner, Br40). Also images with an iterative reconstruction algorithm were reconstructed, with a level 3 iterative strength. Sinogram affirmed iterative reconstruction (SAFIRE, Siemens Healthcare, Forchheim, Germany) was used in the second-generation scanner. Adaptive model-based iterative reconstruction (ADMIRE, Siemens Healthcare, Forchheim, Germany) was used in the third-generation scanner [16–18]. The iterative reconstructions were made to verify whether iterative reconstruction methods influenced the image noise assessment. All images were reconstructed at the maximum field of view available for all pitch values, i.e. 332 mm for the second-generation scanner and 354 mm for the third-generation scanner.

Noise measurements were performed for each reconstructed image throughout the phantom. Measurements of the standard deviation of the CT numbers in a homogeneous region of interest were performed with mathematical computing software (MATLAB R2008a, The MathWorks Inc., Natick, Massachusetts, USA). It was assumed that a constant image noise level as a function of the longitudinal position of the image corresponds to a constant slice thickness equal to the nominal thickness throughout the reconstructed volume.

## Results

### Collimator efficacy

**Fig. 1:**
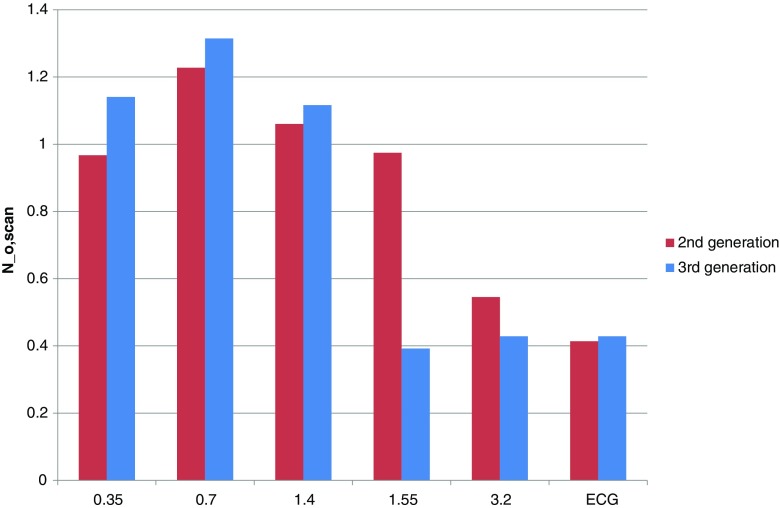
Number ${N}_{\mathrm{o},\mathrm{scan}}$ of additional rotations as a function of pitch for both generations of DSCT scanner. Values labeled with “ECG” correspond to a pitch of 3.4 and 3.2 for the second- and third-generation scanner, respectively. A substantial difference between scanner generations is present at pitch 1.55 only

Figure 1 shows the number ${N}_{\mathrm{o},\mathrm{scan}}$ as a function of pitch for both generations of DSCT scanner. This number is slightly higher than 1 for pitches less than 1.55. For pitches of 1.55 or more, this number drops to approximately 0.4. No large differences between the second- and third-generation DSCT scanner were observed, with the exception of the considerably lower number of extra rotations at pitch 1.55 for the third-generation scanner.

**Fig. 2:**
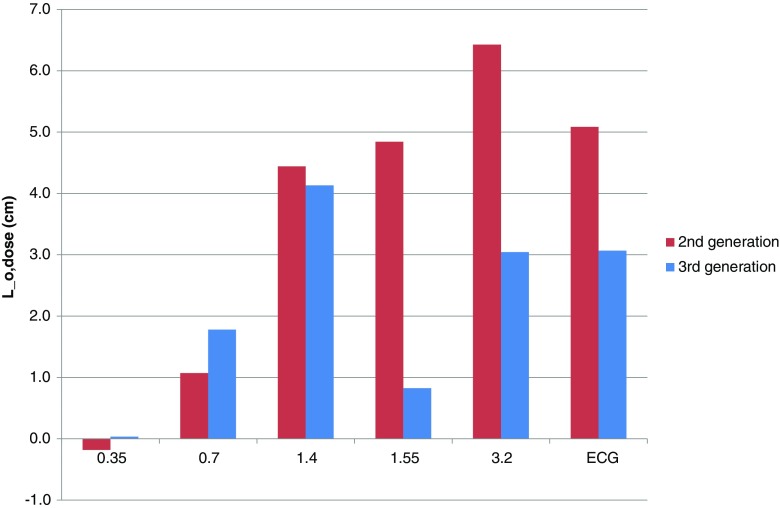
Effective overranging length ${L}_{\mathrm{o},\mathrm{dose}}$ as a function of pitch for both generations of DSCT scanner. Values labeled with “ECG” correspond to a pitch of 3.4 and 3.2 for the second- and third-generation scanner, respectively. ${L}_{\mathrm{o},\mathrm{dose}}$ is up to 4 cm shorter for the third-generation DSCT scanner than for the second-generation DSCT scanner

Figure 2 shows the effective overranging length ${L}_{\mathrm{o},\mathrm{dose}}$ as a function of pitch for both generations of DSCT scanner. The length is comparable for the second- and third-generation DSCT scanner for pitches less than 1.55 (differences less than 0.8 cm). In these cases, the overranging length is virtually absent at pitch 0.35 and increases with increasing pitch. For pitches of 1.55 or more, the length is 2–4 cm lower for the third-generation DSCT scanner than for the second-generation DSCT scanner.

**Fig. 3:**
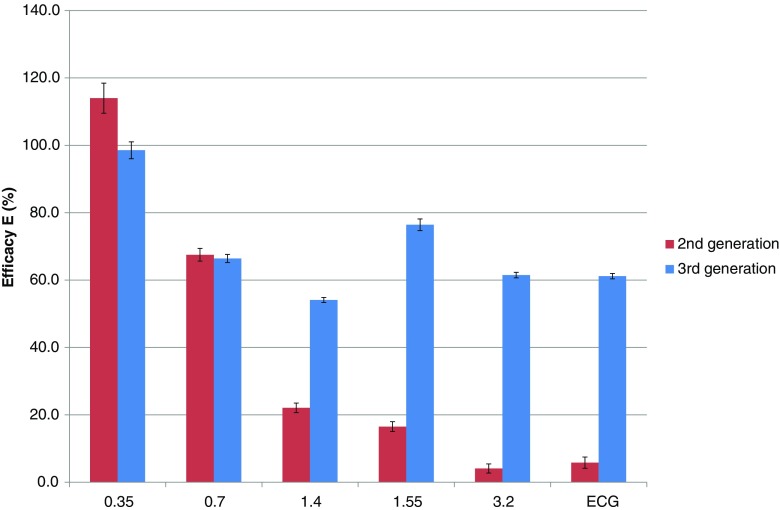
Efficacy *E* as a function of pitch for both generations of DSCT scanner. Values labeled with “ECG” correspond with a pitch of 3.4 and 3.2 for the second- and third-generation scanner, respectively. For the second-generation scanner, efficacy can be as low as 10%. For the third-generation scanner, efficacy lies above 50% for all pitch values. The *error bars* illustrate the uncertainty in *E* as estimated by propagation of the uncertainties in the underlying variables

Figure 3 shows the efficacy *E* as a function of pitch for both generations of DSCT scanner. For the second-generation scanner, efficacy is high at low pitch and rapidly decreases to approximately 10% at the maximum pitch. For the third-generation scanner, efficacy is high at low pitch as well and remains above 50% for higher pitch values.

The results in Figs. 1, 2, and 3 are for a scan length of 300 mm. For scan lengths of 100 mm and 200 mm, the efficacy values did not change more than 1 percentage point compared to the corresponding values at a length of 300 mm, except for the third-generation scanner at pitches of at least 1.55 and a scan length of 100 mm. In these cases the efficacy was 46–65% instead of 61–76%.

### Image noise measurements

**Fig. 4:**
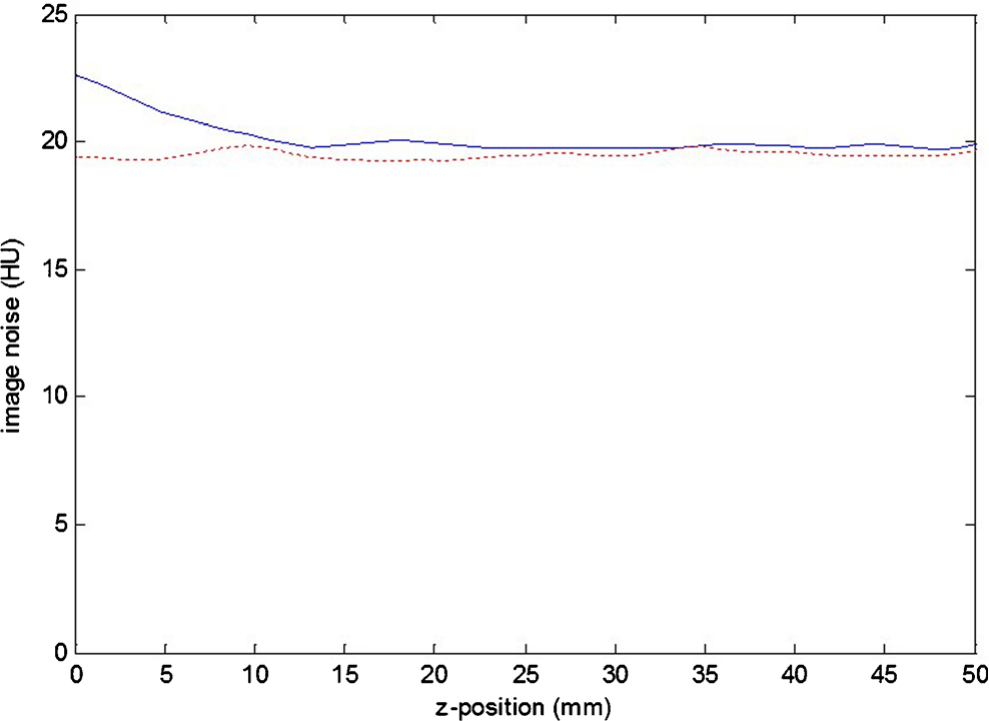
Standard deviation of image noise as a function of *z* position for the second-generation scanner (*dashed red line*) and third-generation scanner (*solid blue line*). Position *z* = 0 mm corresponds to the first position that can be reconstructed. Scan and reconstruction parameters: pitch 1.55; kernel B30f (second-generation) and Br40 (third-generation); slice thickness 10 mm

In general, measured image noise varied less than 1 HU as a function of the *z* position of the reconstructed slice. In Fig. 4 a typical example of constant image noise is shown for the second-generation scanner at pitch 1.55 (dashed red line), scan length of 300 mm and a reconstructed slice thickness of 10 mm. There were three exceptions to this flat noise profile: a slight noise increase was present at the edge of the imaged volume for the third-generation scanner at pitch 1.55 (see solid blue line in Fig. 4). Such an increase was present at pitch 0.35 for both scanners as well (not shown).

The noise increase at the edge of the imaged volume is assumed to be the result of the smaller amount of data and thus dose used for image reconstruction of the corresponding slices compared to the amount of data used for reconstruction of the more centrally located slices in the volume. Consequently, the effective slice thickness at the edges of the imaged volume might be smaller than the nominal thickness of 10 mm.

In cases in which iterative reconstruction techniques were used, the image noise level decreased, as expected. The reconstruction technique and the reconstructed slice thickness did not affect the shape of the image noise profiles as a function of slice position.

## Discussion

The improved performance of the third-generation DSCT scanner over the second-generation DSCT scanner with respect to the reduction of overranging dose in spiral CT for the full range of pitch values was investigated and quantified.

At least two overranging dose reduction strategies exist. One can reduce the number of rotations required for image reconstruction of the very first and last slice of the imaged volume or one can block the radiation that is not used for image reconstruction with the aid of a dynamic collimator. Both strategies are applied in the DSCT scanners investigated.

For a given pitch value, the number of overranging rotations was approximately equal for the second- and third-generation scanner (see Fig. 1), except at pitch 1.55 where the third-generation scanner used approximately half the number of overranging rotations compared to the second-generation scanner. Consequently, the overranging dose showed the largest relative change at this pitch value (see Fig. 2). In general, however, overranging dose was reduced by improved performance of the dynamic collimator. Note that a relatively low efficacy can be due to the particular reconstruction algorithm used and not technical limitations of the dynamic collimator. Overranging might therefore be even further reduced by dedicated reconstruction techniques that are able to reconstruct images beyond the boundaries of the currently imaged volume [19]. Connected to this issue, it would be impossible to gain an efficacy of 100% for high pitch scan mode because of incomplete sampling and arising artefacts, which would deteriorate image quality.

As a result of the inclusion of the nominal slice width (10 mm) in the definition of the length of the reconstructable volume and thus in the definition of the effective overranging length and efficacy, some paradoxical results were present at pitch 0.35: the effective overranging length had a negative value and the efficacy was higher than 100% for the second-generation scanner. Given the exceptional noise behaviour at pitch 0.35, an explanation can be found in the fact that the effective slice thickness of the first and last slice most likely was smaller than the nominal value. This leads to an overestimation of the length *L*_r_ and an underestimation of the overranging dose. Similarly, the efficacy for the third-generation scanner at pitch 0.35 and at pitch 0.7 might be lower than reported as a result of the possibly reduced effective slice thickness and overestimated volume length *L*_r_.

For high pitch values and a short scan length of 100 mm, the efficacy was less than at long scan lengths of 200 mm and 300 mm. This can be explained by the additional slot plate with a fixed opening taking over in dynamic collimation in dual source mode. The slot plate is designed to fully perform the opening phase, even if the trigger for the closing phase is received during the opening phase. For short scans, the time needed for fully opening and fully closing may be longer than the total scan time. Hence, at the end of a short scan, the collimator may not be fully closed. However, these conditions are very rarely met clinically.

Even with a perfectly working dynamic collimator, the dose penalty will increase with increasing pitch. This is because the scanner keeps the effective mAs constant when increasing the pitch by increasing the tube current [11]. Consequently, the overranging dose will increase although the number of additional rotations does not change. This effect can nicely be seen in Fig. 1 where the number of additional rotations does not change when changing the pitch from 1.55 to 3.2 in a third-generation scanner while in Fig. 2 the effective overranging length does increase in this case.

Nevertheless, this study showed an improved performance of the dynamic collimator in the third-generation DSCT scanner at high pitch mode. This is clinically relevant information to determine optimal scan protocols. When high scan speeds are preferred as a result of non-cooperative patients (e.g. newborns), it is useful to know that with the dual-source, high pitch scan modes the dose penalty can be less than the penalty in single-source, low pitch mode thanks to the combination of an effectively working dynamic collimator at high scan speeds and the smaller number of additional rotations made in dual source mode.

## Conclusion

Thanks to dynamic collimation, approximately 50% or more of the overranging dose is blocked in the latest generation DSCT scanner. In comparison to the second-generation scanner, the improved dynamic collimator is better capable of shielding the overranging dose, especially in the high pitch, high speed scan modes.

## Abbreviations and acronyms

ADMIRE: advanced modeled iterative reconstruction
CT: computed tomography
CTDI: computed tomography dose index
DLP: dose–length product
DSCT: dual source computed tomography
ECG: electrocardiogram
RDSR: radiation dose structured report
SAFIRE: sinogram affirmed iterative reconstruction
SSCT: single source computed tomography
SSP: slice sensitivity profile

## References

[1] Irwan R, de Vries HB, Sijens PE (2008). The impact of scan length on the exposure levels in 16- and 64-row multidetector computed tomography: a phantom study. Acad Radiol.

[2] Schilham A, van der Molen AJ, Prokop M, de Jong HW (2010). Overranging at multisection CT: an underestimated source of excess radiation exposure. Radiographics.

[3] Theocharopoulos N, Damilakis J, Perisinakis K, Gourtsoyiannis N (2007). Energy imparted-based estimates of the effect of z overscanning on adult and pediatric patient effective doses from multi-slice computed tomography. Med Phys.

[4] Kroft LJ, Roelofs JJ, Geleijns J (2010). Scan time and patient dose for thoracic imaging in neonates and small children using axial volumetric 320-detector row CT compared to helical 64-, 32-, and 16- detector row CT acquisitions. Pediatr Radiol.

[5] Tzedakis A, Damilakis J, Perisinakis K, Stratakis J, Gourtsoyiannis N (2005). The effect of z overscanning on patient effective dose from multidetector helical computed tomography examinations. Med Phys.

[6] Trevisan D, Bonutti F, Ravanelli D, Valentini A (2014). Real time evaluation of overranging in helical computed tomography. Phys Med.

[7] van der Molen AJ, Geleijns J (2007). Overranging in multisection CT: quantification and relative contribution to dose–comparison of four 16-section CT scanners. Radiology.

[8] Tzedakis A, Damilakis J, Perisinakis K, Karantanas A, Karabekios S, Gourtsoyiannis N (2007). Influence of z overscanning on normalized effective doses calculated for pediatric patients undergoing multidetector CT examinations. Med Phys.

[9] Tzedakis A, Perisinakis K, Raissaki M, Damilakis J (2006). The effect of z overscanning on radiation burden of pediatric patients undergoing head CT with multidetector scanners: a Monte Carlo study. Med Phys.

[10] Tsalafoutas IA (2011). The impact of overscan on patient dose with first generation multislice CT scanners. Phys Med.

[11] Christner JA, Zavaletta VA, Eusemann CD, Walz-Flannigan AI, McCollough CH (2010). Dose reduction in helical CT: dynamically adjustable z-axis x-ray beam collimation. AJR Am J Roentgenol.

[12] Deak PD, Langner O, Lell M, Kalender WA (2009). Effects of adaptive section collimation on patient radiation dose in multisection spiral CT. Radiology.

[13] Shirasaka T, Funama Y, Hayashi M, Awamoto S, Kondo M, Nakamura Y (2012). Reduction of the unnecessary dose from the over-range area with a spiral dynamic z-collimator: comparison of beam pitch and detector coverage with 128-detector row CT. Radiol Phys Technol.

[14] National Electrical Manufacturers Association (2011). Digital imaging and communications in medicine (DICOM) Part 16: content mapping resource.

[15] Commision iE (2012) European standard IEC 60601-2-44 Amendment 1

[16] Gordic S, Morsbach F, Schmidt B, Allmendinger T, Flohr T, Husarik D (2014). Ultralow-dose chest computed tomography for pulmonary nodule detection: first performance evaluation of single energy scanning with spectral shaping. Invest Radiol.

[17] Wang R, Schoepf UJ, Wu R, Reddy RP, Zhang C, Yu W (2012). Image quality and radiation dose of low dose coronary CT angiography in obese patients: sinogram affirmed iterative reconstruction versus filtered back projection. Eur J Radiol.

[18] Winklehner A, Karlo C, Puippe G, Schmidt B, Flohr T, Goetti R (2011). Raw data-based iterative reconstruction in body CTA: evaluation of radiation dose saving potential. Eur Radiol.

[19] Tang X, Hsieh J, Dong F, Fan J, Toth TL (2008). Minimization of over-ranging in helical volumetric CT via hybrid cone beam image reconstruction–benefits in dose efficiency. Med Phys.

